# Analysis of Machine Learning Algorithms for Anomaly Detection on Edge Devices

**DOI:** 10.3390/s21144946

**Published:** 2021-07-20

**Authors:** Aleks Huč, Jakob Šalej, Mira Trebar

**Affiliations:** Faculty of Computer and Information Science, University of Ljubljana, Večna Pot 113, SI-1000 Ljubljana, Slovenia; aleks.huc@fri.uni-lj.si (A.H.); js4466@student.uni-lj.si (J.Š.)

**Keywords:** machine learning, classification, edge computing, imbalanced dataset, training dataset, anomaly detection, clustering

## Abstract

The Internet of Things (IoT) consists of small devices or a network of sensors, which permanently generate huge amounts of data. Usually, they have limited resources, either computing power or memory, which means that raw data are transferred to central systems or the cloud for analysis. Lately, the idea of moving intelligence to the IoT is becoming feasible, with machine learning (ML) moved to edge devices. The aim of this study is to provide an experimental analysis of processing a large imbalanced dataset (DS2OS), split into a training dataset (80%) and a test dataset (20%). The training dataset was reduced by randomly selecting a smaller number of samples to create new datasets *Di* (*i* = 1, 2, 5, 10, 15, 20, 40, 60, 80%). Afterwards, they were used with several machine learning algorithms to identify the size at which the performance metrics show saturation and classification results stop improving with an F1 score equal to 0.95 or higher, which happened at 20% of the training dataset. Further on, two solutions for the reduction of the number of samples to provide a balanced dataset are given. In the first, datasets *DRi* consist of all anomalous samples in seven classes and a reduced majority class (‘NL’) with *i* = 0.1, 0.2, 0.5, 1, 2, 5, 10, 15, 20 percent of randomly selected samples. In the second, datasets *DCi* are generated from the representative samples determined with clustering from the training dataset. All three dataset reduction methods showed comparable performance results. Further evaluation of training times and memory usage on Raspberry Pi 4 shows a possibility to run ML algorithms with limited sized datasets on edge devices.

## 1. Introduction

Cloud, fog, edge, and mist computing are, more or less, well known paradigms introduced in the Internet of Things (IoT) [1]. Lately, by keeping processing closer to the edge of the network, many issues such as low latency, privacy, and location awareness requirements can be mitigated, with the added benefit of increased privacy as raw data are not sent to the cloud. To that end, the authors provide an overview of fog computing and other related paradigms, such as edge computing, mist computing, and mobile computing. Sometimes, the distinctions between these computing paradigms are not yet clear, which gives additional opportunities for further research into the current landscape where sensor and other IoT devices are located.

In recent years, the Internet of Things (IoT) has been a rapidly growing network of devices, which generate a massive amount of data [2]. Cloud computing is suitable in some IoT sectors but is unsuitable and impractical in connected sensor systems in homes, cities, and industries. Often, sending data to remote systems leads to privacy problems, network bandwidth decrease, and even loss of data. In fact, for many reasons, the need arises to perform the computing close to the data source. It is also important to incorporate intelligence and enable adaptations to rapid changes and unexpected events.

Classifying and surveying current edge computing architectures and platforms provides a review, which reveals that the edge of the network is not always clearly defined [3]. Three categories could be used to define functionalities: (i) resource-rich servers deployed at the edge; (ii) heterogeneous edge nodes; and (iii) edge-cloud federation. To show some advantages of edge computing for IoT applications, the second part of the article focuses on mobile gaming as a use case. Placing limited computing resources closer to the network edge improved the quality of service by reducing latency and improving the overall responsiveness of the game.

Deep learning is widely used and has been very successful across various application domains [4]. Based on the extended review, it is recognized as a solution to the need for processing data on edge devices, mainly to reduce latency, but with the added benefits of privacy, bandwidth efficiency, and scalability. The main areas of deep learning applications are computer vision, natural language processing (NLP), and network functions, including intrusion detection. The complexity and high resource requirements of deep neural networks (DNNs) are forcing the development of solutions with different optimizations in terms of model design, model compression, and dedicated hardware solutions.

With the development of IoT applications in cyber-security, traffic inspection and classification tasks are moved to edge devices in order to implement processing intelligence near the data source [5]. The pre-build classification models are effectively run on devices, while some heavy computational tasks of training data are performed in cloud-based architectures. In the implementation of an automatic attack detection system, an idea of a cloud-assisted extreme learning machine (ELM) classifier showed the efficient computation and analysis of collected data.

Anomaly detection is one of the challenges in securing wireless sensor networks (WSN) [6]. It is important to reduce false alarms. For this purpose, machine learning algorithms have become very useful and widely used. Very often, they require the dataset in the node to perform training and evaluation. The authors propose an online locally weighted projection regression (OLWPR) model for anomaly detection in WSN, where predictions are only performed by local functions on a subset of data to provide low computation complexity. The comparison of the OLWPR with existing methods (logistic regression (LR), random forest (RF), AdaBoost, decision tree (DT), support vector machine (SVM), artificial neural network (ANN)) showed better results in terms of accuracy, F1 score, average RMSE, and average percentage error.

Another anomaly detection problem was analyzed using machine learning to predict anomalies and attacks on IoT systems [7]. The supervised learning algorithms LR, SVM, DT, RF, and ANN detect anomalies in the DS2OS dataset. The evaluation metrics used in the performance comparison were accuracy, precision, recall, F1 score, confusion matrix, and area under the receiver operating characteristic (ROC) curve.

To detect anomalies, i.e., features with abnormal behavior in the imbalanced data, an approach based on a long short-term memory (LSTM) auto-encoder and one-class support vector machine (OC-SVM) is proposed [8]. The LSTM auto-encoder is trained to learn the normal traffic pattern and compressed representation of the input data. The OC-SVM then detects anomaly-based attacks. The experiments on the latest dataset (InSDN) of intrusion detection systems (IDSs) for software-defined networks show that the proposed model provides a high detection rate of malicious traffic and significantly reduces the processing time.

Anomaly detection has been an active area of research for several years. Recently, the deep learning approach has gained wide acceptance. Many studies in journals and conferences provide an overview of the progress in various solutions based on machine learning and data mining [9]. The authors give a comprehensive overview of problem complexity, representative open source algorithms, pre-training models, and architectures.

Many devices in the IoT can be problematic due to the lack of resources and overlooked integrated security [10]. An increased number of intrusion attacks can cause many faults and existing intrusion detection systems cannot provide sufficient protection. The article highlights the comparison of state-of-the-art machine learning algorithms (k-nearest neighbor (KNN), SVM, DT, RF, ANN) for binary and multiclass classification on Bot-IoT datasets.

Most of the edge devices, on which ML algorithms are expected to run, have limited memory size and computing power, which requires a deeper understanding of the data. A detailed review of models, architectures, and requirements provides an insight into the execution of ML algorithms on large imbalanced datasets.

The main contributions of the presented research paper are:

- Detailed analysis of five ML algorithms (logistic regression, support vector machine, decision tree, random forest, and artificial neural network) for determination of anomaly detection performance on traffic traces between different IoT nodes communicating over DS2OS common middle-ware.
- Proposal of two general and intuitive approaches for keeping comparable classification results and reducing the size of an imbalanced training dataset by randomly under-sampling the majority class (‘NL’), and by under-sampling each class with clustering and selecting the most representative observation samples.
- Evaluation of ML algorithms training times on Raspberry Pi 4 comparing small randomly specified imbalanced datasets and new reduced balanced datasets, as well as examining the results of memory usage for suitable implementation on resource-constrained edge devices.

The paper is organized as follows. In Section 2, we present the edge computing, the machine learning algorithms, and most relevant evaluation metrics. Section 3 shows the details of an imbalanced dataset with various anomalies, an explanation of two proposed determinations of balanced training datasets, experimental results, and detailed performance analysis of determined datasets. As an edge device, Raspberry Pi 4 was selected to train and run local machine learning algorithms for the evaluation of the computation time and the memory usage. Section 4 draws a conclusion.

## 2. Related Work

### 2.1. Edge Computing

An edge device is any device with limited computation, memory, and energy resources, which is constrained and not easy to upgrade. Usually, it is not possible to add more memory or battery power without replacing the old device with a new one, which leads to additional costs. This leads to new solutions where the use of machine learning algorithms or suitable preprocessing of raw data could be implemented.

Sensor technologies enable numerous challenges in data acquisition, storage, and processing. Environmental data are subject to an incompleteness that must be acquired and processed in real-time [11]. The presented solution to this problem was an environmental gas detection monitoring system implemented on edge devices where sensor data enter the anomaly detection module (ADM) to determine if the given data are an anomaly based on the trained model. By using the system on the edge device, a hazardous gas condition can be quickly detected and action taken. It also provides more robust prediction results with verified data.

Another example of anomaly detection is rare-event detection system, which uses unsupervised learning on the IoT edge [12]. This system runs an AGILE gateway framework on Raspberry Pi and uses a connected USB microphone to detect rare events, such as gunshot, glass break, scream, and siren with 90% precision and recall. By leveraging two-staged unsupervised machine learning strategy, rare events were detected without any prior knowledge. Authors argue that results such as these prove that edge and fog paradigms could be viable solutions, especially for the cases where time is critical and data processing near the source is highly required.

The use of machine learning (ML) algorithms provides various solutions on edge devices, but they are restricted by computational capabilities [2]. It is important to measure the accuracy on a dataset large enough to confirm the obtained results as valid and to select an optimal choice of hardware. Microcontrollers can be used in IoT applications to run neural networks with a pre-trained model using libraries optimized for the small size of memory. The proposed hardware used for IoT edge devices to run a deep neural network (DNN) model, like support vector machine (SVM), convolutional neural network (CNN), and logistic regression (LR), include Raspberry Pi model 3 (ARM v8) [13,14], STM32F401RE (ARM Cortex—M4) [2], and ESP 32 [15]. Based on the detailed review, the latest development of edge computing provides deployment of ML algorithms to satisfy the requirements of privacy, energy consumption, and computational complexity.

The performance of clusters of low-resource devices for ML tasks has been studied in a distributed architecture in tourism applications based on big data [16]. The Raspberry Pi micro-cluster was configured with industry-standard platforms to evaluate local training of ML algorithms and execution of ML-based predictions. The distributed architecture demonstrates the ability to run a small cluster as an edge computing application to determine the data loading, the model training, and prediction tasks. Analysis of the size of the dataset and the speed of model training shows the expected performance tradeoff.

Many studies raise the question of whether IoT data should be sent directly to the cloud or whether pre-processing should be implemented at the network edge and only the necessary data should be sent [17]. Anomaly detection is presented as an edge mining technique to reduce the transmission overhead when the frequently monitored activities contain sparse set of anomalies. The authors present benchmark results for four ML classifiers (random forest, multilayer perceptron, K-nearest neighbor, and discriminant analysis) on a Raspberry Pi 3 edge device for different anomaly scenarios. K-nearest neighbors showed reliable prediction accuracy but requires excessive overhead with time and energy loss on the edge device, while the others can save time and energy on the edge device during data transmission.

Edge and cloud computing are presented as smart manufacturing in industrial and other implementations of sensing and control monitoring [18]. On both, the benchmarking of latencies uses performance metrics of times to analyze an IoT-based machinery monitoring-based study. A Raspberry Pi 3 runs a Python program to acquire data, compute the time domain features, and fast-Fourier transform (FFT). The evaluation results show that the choice between edge and cloud implementation depends on the computation time of the algorithm and overheads related to data acquisition.

The prototype of a bracelet with sensors helps detect anomalies in users’ daily life or situations at risk [19]. The complete system consists of sensors and electronic components for monitoring vital signs and detecting anomalies. Combining multiple modules for different measurements, a Raspberry Pi, model 3B, serves as a concentrator node on the Ubuntu Mate operating system. It collects data and provides a system with a Mosquito server. The data were sent to an NVIDIA Jetson Nano device, which enables in-hardware processing and edge computing.

Edge anomaly detection for sensor networks appears in many research areas of the IoT in industrial solutions [20]. Many traditional clustering methods, such as K-means and C-means have been proposed for data analysis and prediction, but they are not directly useful for IoT applications in underground mining systems. An edge computing model with anomaly detection algorithms was proposed for sensor nodes to collect and pre-process data and then detect anomalies on sink nodes. Performance analysis on the experimental platform showed acceptable accuracy, delay, and energy consumption in the required environment.

### 2.2. Machine Learning

Machine learning (ML) is a subset of artificial intelligence (AI) where the collected dataset is used to build a model, which can make predictions on completely new data. By using AI on the IoT, the new field of Artificial Intelligence of Things (AIoT) emerged with the solutions of running ML algorithms remotely on physical devices [21]. It delivers intelligent and connected systems, like wearable devices, digital assistants, smart sensors, vehicle industry, healthcare, manufacturing, supply chain, and others. Currently, IoT devices collect and process data to react to an event by presenting the facts. In the future, the aim of AIoT systems will be to detect events and failures and automatically take an action and become a brain of the connected systems.

Lately, IoT devices in connection with machine learning (ML) have provided solutions for smart transportation [22]. The authors reviewed the possibilities of systems that locally analyze data streams from their sensors in real time. Many proposed infrastructures consist of three groups of nodes with applied ML algorithms: (i) the IoT nodes at the edge of the network to support collection and exchange of data; (ii) the fog nodes to offer computing and storage of data; and (iii) cloud nodes to handle advanced tasks with intensive data analysis, and run application software. Such systems lead to the enhanced capabilities of applications, such as route optimization, accident prevention, and detection of road anomalies in use cases of smart cities where the IoT has been implemented. Additionally, the review showed a lack of techniques to further enhance the intelligence in applications on city traffic data, which would help to explore the best solutions.

Intrusion detection systems are one of the key elements of the increasing security threats. Managing a large amount of unnecessary features using machine learning proved to be one of the best ways to design an intrusion detection system [23]. A hybrid solution using decision tree (DT) for feature selection and support vector machine (SVM) for prediction improved the true positive and false positive criteria and accuracy compared to the best previously published works.

The problem of anomaly detection in a distributed network of nodes was successfully studied using a novel support vector machine (SVM)-based approach [24]. The conventional SVM algorithm was reformulated to achieve reduced communication load and computational complexity through distributed and efficient gradient-based training to obtain an estimate for the separating hyperplane parameters.

#### 2.2.1. ML Algorithms

Five machine learning (ML) algorithms were considered in the study: logistic regression (LR), support vector machine (SVM), decision tree (DT), random forest (RF), and artificial neural network (ANN). They are forms of supervised learning where from the available input-output pairs a model is built by using an algorithm to learn the mapping function from the input to the output. The main goal is that the mapping function approximates well on new input data to predict the output values. ML algorithms were implemented in Python using scikit-learn and Keras libraries [25]. For dataset storing and manipulation, a Pandas framework was used. The ML algorithms are:

- Logistic regression (LR) is a linear model for classification [26]. In the scikit-learn implementation used, regularization is applied by default in Python as a function call *LogisticRegression(class_weight = ’balanced’, max_iter = 10,000, n_jobs = −1)*. The solver for the optimization problem is *lbfgs* [27]. By default, it uses the cross-entropy loss in a multiclass case. The parameter *class_weight* was set to balanced mode, which uses values of output y to automatically adjust weights inversely proportional to class frequencies in the input data. The parameter *max* sets the maximum number of iterations for the solvers to converge and was raised from the default value of 100 to 10,000 to prevent solvers from not converging. The parameter *n_jobs* sets the number of CPU cores that can be used in case of a multiclass problem with a one-vs.-rest (OvR) scheme and was set to −1 for all runs (−1 means using all available processors), although it had no effect in this case as cross-entropy loss was used for the multiclass problem.
- Support vector machine (SVM) is a supervised learning model used for classification and regression [28]. Scikit-learn’s C-Support Vector Classification implementation is based on libsvm defined as function call *SVC(class_weight = ‘balanced’)*. By default, it uses a Radial-Basis-Function kernel and l2 regularization with the strength of 1.0 [29]. The multiclass support is handled according to a one-vs.-one scheme. The parameter *class_weight* was set to balanced mode, which uses values of y to automatically adjust weights inversely proportional to class frequencies in the input data.
- Decision tree (DT) is a non-parametric supervised learning method for classification [30]. In the scikit-learn implementation used, it is defined as *DecisionTreeClassifier()* [31], with default criterion for measuring the quality of a split using Gini impurity. This is a measure of how often a randomly chosen element from the set would be incorrectly labeled. No parameters were set outside of their default values.
- Random forest (RF) is one of the ensemble methods which combines the predictions of several base estimators to improve the robustness of the estimator [32]. Each tree in the ensemble is built from a sample drawn with a replacement from the training set. By default, in the function call *RandomForestClassifier(n_estmators = 100, n_jobs = −1),* there are 100 trees in the scikit-learn implementation of the algorithm, with Gini impurity as a default measure of split’s quality. The whole dataset is used to build each tree. The parameter *n_jobs* was set to −1 to use all available CPU cores for parallelizing fit and predicted methods over the trees.
- Artificial neural network (ANN) is a circuit of connected neurons that each deliver outputs based on their inputs and used predefined activation functions [33]. A *Keras* library with *Tensorflow* backend was used for the ANN training model with 11 input nodes on the input layer, 32 nodes on a hidden layer with *relu* (rectified linear) activation function, and 8 output nodes with *softmax* activation function to normalize the outputs. The selected optimization function was the Adam optimizer. The loss function was *sparse categorical cross entropy* and the number of epochs was set to ten.

#### 2.2.2. Evaluation Metrics

**Figure 1:**
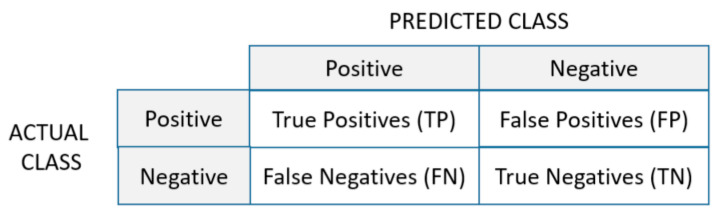
Confusion matrix.

The comparison of learning algorithms for the presented approaches of generated subsets from the original dataset was based on standard performance metrics for evaluating classification models. They were calculated from results of true positive (TP), false positive (FP), false negative (FN), and true negative (TN) for multiple classes. For example, the definitions of TP, FP, FN, and TN for Ci are: (i) TP(Ci)—all the instances of Ci that are classified as Ci; (ii) FP(Ci)—all the non-Ci instances that are classified as Ci; (ii) FN(Ci)—all the Ci instances that are not classified as Ci; (vi) TN(Ci) = all the non-Ci instances that are not classified as Ci. The classification metrics from *sklearn.metrics* in Python are [34]:

- Accuracy determines how many predictions the classifier got right from all the predictions (Equation (1)). It is defined as a sum of number of true positives (TP) and true negatives (TN) divided with the sum of number of true positives (TP), true negatives (TN), false positives (FP), and false negatives (FN):
  (1) $$\mathrm{Accuracy}\;=\frac{\mathrm{TP}+\mathrm{TN}}{\mathrm{TP}+\mathrm{TN}+\mathrm{FP}+\mathrm{FN}}$$
  While the higher the number the better in case of an approximately equal number of samples in all classes, accuracy alone often leads to an error in the classification of the minor class in imbalanced datasets;
- Precision is the fraction of relevant instances among the retrieved instances (Equation (2)). It is defined as a number of true positive (TP) results divided by the number of true positive (TP) results and false positive (FP) results;
  (2) $$\mathrm{Precision}\;=\frac{\mathrm{TP}}{\mathrm{TP}+\mathrm{FP}}$$
- Recall is the fraction of the total amount of relevant instances that were actually retrieved (Equation (3)). It is defined as a number of true positive (TP) results divided by true positive (TP) results and false negative (FN) results;
  (3) $$\mathrm{Recall}\;=\frac{\mathrm{TP}}{\mathrm{TP}+\mathrm{FN}}$$
- F1 score is the harmonic mean of precision and recall (Equation (4)). The highest possible value of F1 is 1, indicating perfect precision and recall, and the lowest possible value is 0, if either the precision or the recall is zero;
  (4) $$F1\;\mathrm{score}\;=\frac{2*\left(\mathrm{Precision}*\mathrm{Recall}\right)}{\left(\mathrm{Precision}+\mathrm{Recall}\right)}$$
- Confusion Matrix is a specific table layout meant to visualize the performance of an algorithm, typically one from a group of supervised learning algorithms. In Python implementation, each row of the matrix represents the instances in an actual class while each column represents the instances in a predicted class (Figure 1). It is easy to see all falsely classified samples. The more samples found on the diagonal of the matrix, the better the model is.

## 3. Results

At the beginning, the dataset description and determination of training datasets is presented in tables in detail for:

- Imbalanced training datasets (*Di*)—randomly selected samples from the training set;
- Balanced datasets (*DRi*)—all anomalous classes and randomly selected samples from class ‘NL’;
- Balanced datasets (*DCi*)—selected clusters of representative samples from all classes.

Further, a detailed performance evaluation of ML algorithms for these groups of training datasets and test dataset is given. The detailed analysis is based on the previously described performance metrics, shown as a comparison of results to identify which approach is viable for implementation as edge computing on Raspberry Pi 4. The training model was performed with 5-fold cross validation. Graphs show only the most representative measurement of accuracy, F1 score, and confusion matrixes to give valuable comparison.

Finally, the training time and memory usage on Raspberry Pi 4 are presented for small datasets *Di*, *DRi*, and *DCi* to evaluate the performance of the proposed solutions of ML algorithms for edge computing.

### 3.1. Dataset

The open source dataset DS2OS contains IoT traffic traces from the application layer captured in an IoT environment [35]. It includes data from different types of devices, to be more specific: the source device, destination device, when and what operation was performed, as well as the normality level information regarding whether an event is considered an anomaly or not. To use the dataset, the first step was the preprocessing of raw data. The following steps were applied:

- Removal of corrupted data, unreadable field values;
- Change of NaN values from column ‘NodeType’ to Malicious;
- Replacement of all non-numeric values in column ‘value’ with numeric representations, all missing values in the same column filled with 0;
- Removal of ‘timestamp’ column from the dataset, as it is irrelevant;
- Use of label encoding on all columns except on column ‘values’.

There are 357,941 samples available in the dataset, with 13 features: ‘sourceID’, ‘sourceAddress’, ‘sourceType’, ‘sourceLocation’, ‘destinationServiceAddress’, ‘destinationServiceType’, ‘destinationLocation’, ‘accessedNodeAddress’, ‘accessedNodeType’, ‘operation’, ‘value’, ‘timestamp’, and ‘normality’. Each sample represents access from one node (source) to another node (destination) inside the IoT network. Normality represents the type of access, defined as normal (‘NL’), and as seven anomalous types: Denial of Service (‘DoS’), Scan (‘SC’), Malicious Control (‘MC’), Malicious Operation (‘MO’), Spying (‘SP’), Data Type Probing (‘DP’), Wrong Setup (‘WS’).

The determination of subsets starts with a random split of the original dataset into initial training and test datasets (80–20%). From the initial training dataset, nine training subsets were determined and together with the training dataset were used as imbalanced datasets in the first approach. These subsets consist of samples from all classes with a fixed random generator to provide comparable results. In the next two approaches, smaller subsets are determined for the purpose of performing edge computing where an imbalanced training dataset is transformed into balanced subsets of data. The first presents the reduction of the largest class ‘NL’ with 97.5% of samples. The second shows the use of the clustering method to include in training representative samples from all classes.

#### 3.1.1. Imbalanced Subsets

**Figure 2:**
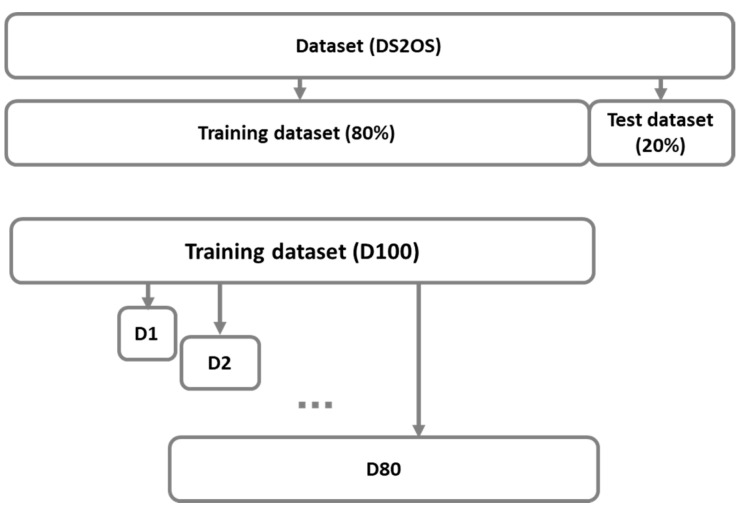
Determination of randomly selected training dataset (80%) and test dataset (20%) from the original dataset (DS2OS), and imbalanced datasets D1, D2, …, D80 from the training dataset (D100).

In our first approach, training datasets consist of randomly selected samples from all classes in previously determined training dataset (Figure 2). Each dataset from D1 to D80 was generated identical between runs, making the results reproducible during many tests.

Table 1 shows class distribution in the training dataset, test dataset, and training subsets. Each subset *Di*, where *i* specifies the percentage of samples, includes randomly selected samples from the original training set.

#### 3.1.2. Random Selection of Class ‘NL’

**Figure 3:**
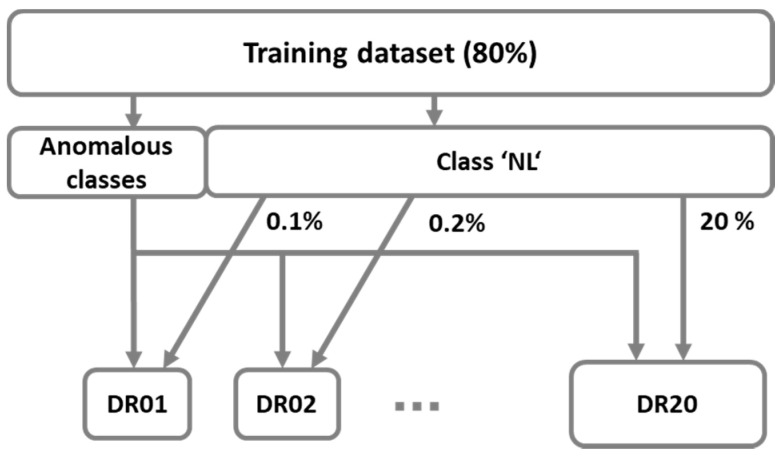
Determination of balanced datasets *DRi* with randomly selected samples from class ‘NL’.

Our first proposed approach of balanced datasets presents the solution of the under-sampling method. The idea is to keep only a part of samples from the majority class. Figure 3 shows datasets that consist of all samples from anomalous classes (‘DoS’, ‘DP’, ’MC’, ’MO’, ’SC’, ’SP’, ‘WS’) and randomly selected samples from the normal class (‘NL’). Additionally, Table 2 shows class distribution in training subsets *DRi*, where *i* specifies the percentage of samples from class ‘NL’.

#### 3.1.3. Subsets of Clusters Data

The main goal of the second approach is to reduce the dataset size by selecting a few of most representative observations while discarding others. We achieve this by clustering the observation of each class separately and then taking the most representative observations. The input to our algorithm is the entire dataset (*D_old_*), the threshold that determines the minimum cluster size (*t*), and the number of representative observations we want to extract (*n*). First, the number of classes presented in the dataset is determined and used for iterating over each class (*c*). Second, observations for class *c* are extracted (*X_c_*) from the entire dataset and input into the DBSCAN clustering algorithm [36].

**Figure 4:**
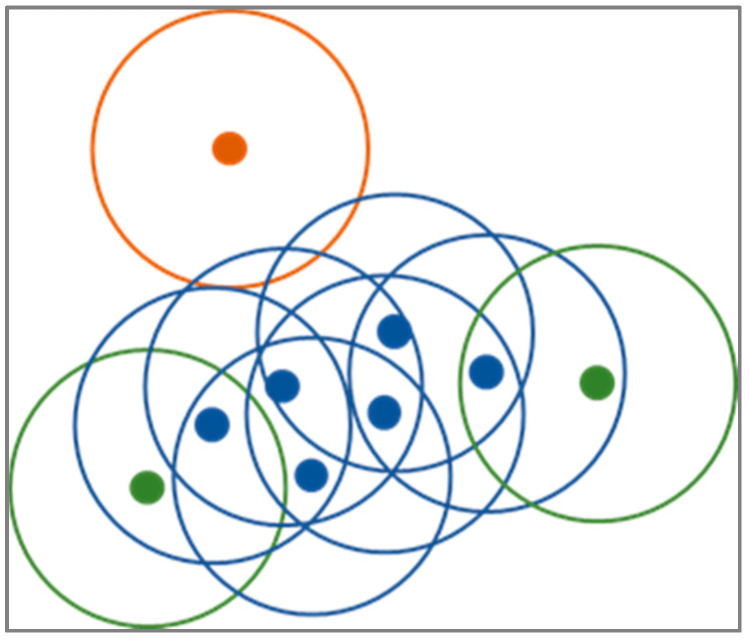
Diagram of DBSCAN clustering procedure with value three for the minimum neighboring points threshold. DBSCAN discriminates between core (blue), non-core (green), and noise (orange) points.

It first finds the points in the neighborhood of every point and those points that have more than a minimum threshold of neighborhood points are determined as core points. Then, clusters are determined by the core points that are in each other’s neighborhoods. All non-core points are then assigned to clusters if they are in the neighborhoods of cluster core points or to noise otherwise (Figure 4). On average, the time complexity of DBSCAN is O(nlogn) and optimized implementation use O(n) of memory. Its main advantages are that it does not require knowing the number of clusters in the dataset in advance, can find clusters of arbitrary shapes, is robust to noise, is determined by only two parameters (i.e., neighborhood size and a minimum threshold of neighboring points), and is able to handle large datasets.

Third, we iterate through the clusters determined by the DBSCAN for class *c*, check their sizes, and ignore those that are smaller than *t* to increase robustness and reduce noise. If the cluster is large enough, then we extract the points of this cluster (*X_p_*) and calculate their centroid with Equation (5):

(5) $$q=\frac{\sum_{i=1}^{k}X_{p_{i}}}{k}$$

Fourth, we calculate the Euclidean distance between the centroid q and each point in *X_p_* (x) with Equation (6):

(6) $$dist\left(q,x\right)=\sqrt{{\left(q_{1}-x_{1}\right)}^{2}+{\left(q_{2}-x_{2}\right)}^{2}+\ldots +{\left(q_{r}-x_{r}\right)}^{2}}$$

Finally, we determine the number of closest points to q, which we add to the reduced dataset (*D_new_*), with the parameter *n* and the size of the cluster. If, for example, the current cluster presents 70% of all points from class *c*, we extract 0.7n of observations from it. So, large clusters provide proportionally more points than small clusters. We repeat these steps first for the remaining clusters and then for the remaining classes. If there are fewer points in the cluster than the number allowed, then we just add all points to the *D_new_*. This property is especially useful when we are dealing with unbalanced datasets, where this approach reduces the number of the majority class observations by selecting only the most representable ones, while keeping all observations of minor classes. The time complexity of this approach is O(mnlogn), where m presents the number of classes and nlogn presents the DBSCAN clustering. This could be improved with the use of more efficient incremental density-based clustering approaches with time complexity O(nm) like DBSCAN++ [37]. On the other hand, the direct clustering of all classes at once and then determining the most representative observations could also prove beneficial; however, handling of a multi-class cluster could be challenging.

**Figure 5:**
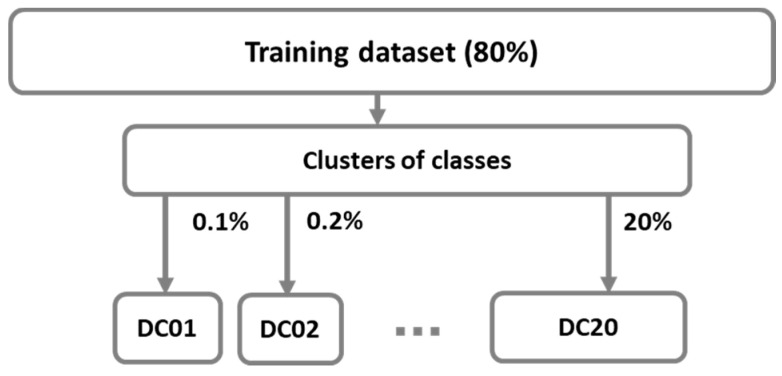
Determination of balanced datasets *DCi* with selection of representative samples using clustering method.

Figure 5 shows two steps in which smaller datasets *DCi* are determined from previously defined clusters as described in the Algorithm 1.
**Algorithm 1:** Dataset reduction using clustering.**Input:***D_old_*, *t*, *n***Output:***D_new_***Function** DatasetReduction(*D_old_*, *t*, *n*): *D_new_* ← [ ]  **for**
*c*
**in** findClasses(*D_old_*) **do**   *X_c_* ← extractClassPoints(*D_old_*,*c*)   *db* ← DBSCAN.fit(*X_c_*)   **for**
*l*
**in**
*db.clusters()*
**do**    *m* ← size(*l*)/size(*X_c_*)    **if**
*m* > *t **do***      *X_p_* ← db.extractClusterPoints(*p*)      *q* ← createCentroid(*X_p_*)    *dist* ← distances(*q*, *X_p_*) *D_new_* ← addClosestNPoints(*D_new_*, *dist*, *X_p_*, *n*)    **end**   **end**  **end****end**

Table 3 shows class distribution in training subsets *DCi*, where *i* specifies the percentage of samples from clusters.

### 3.2. Evaluation of Imbalanced Training Datasets

Imbalanced training datasets *Di* provide general results for selected evaluation metrics to recognize the smallest randomly selected dataset that gives results, which are slightly, or not at all improved with a larger number of samples.

#### 3.2.1. Classification Results

**Figure 6:**
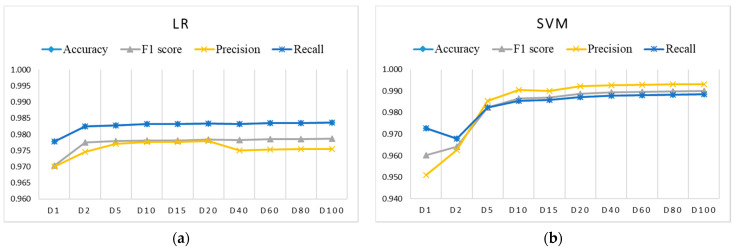
Evaluation results on test dataset for imbalanced training datasets *Di* using: (**a**) LR (coincidence of accuracy and recall); (**b**) SVM (coincidence of accuracy and recall).

**Figure 7:**
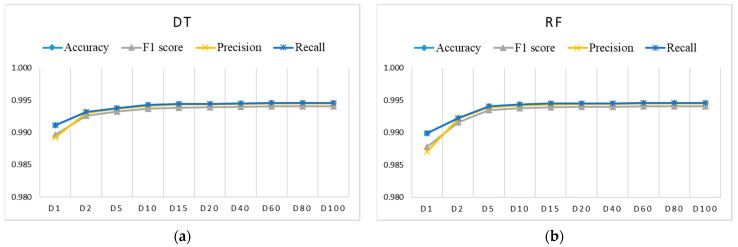
Evaluation results for imbalanced training datasets *Di* using: (**a**) DT (coincidence of accuracy, recall, and precision only for datasets larger than D1); (**b**) RF (coincidence of accuracy, recall, and precision only for datasets larger than D1).

**Figure 8:**
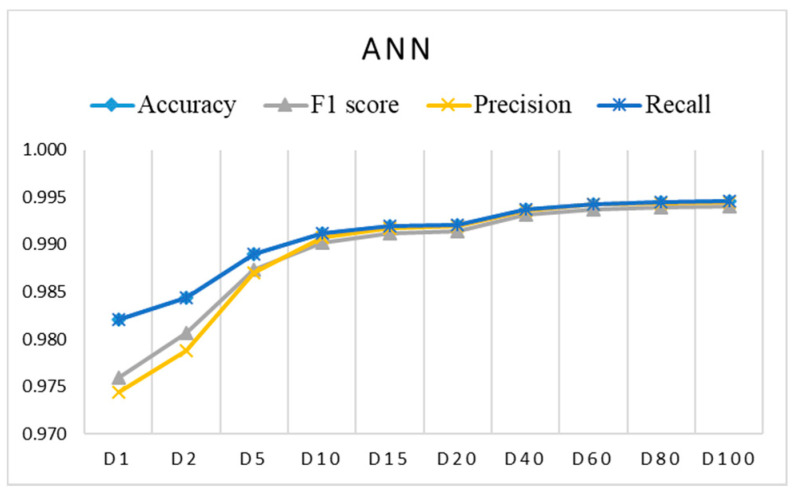
Evaluation results for imbalanced training datasets *Di* using ANN (coincidence of accuracy and recall).

Figure 6, Figure 7 and Figure 8 show the evaluation results for training datasets *Di* (D1, D2, D5, D15, D20, D40, D60, D80, D100) obtained on the test dataset. The LR, DT, and RF algorithms perform similarly and already reach almost the best performance with small datasets D2, D5, and D10. The SVM and ANN algorithms perform slightly worse and reach the best performance gradually with larger training datasets.

**Figure 9:**
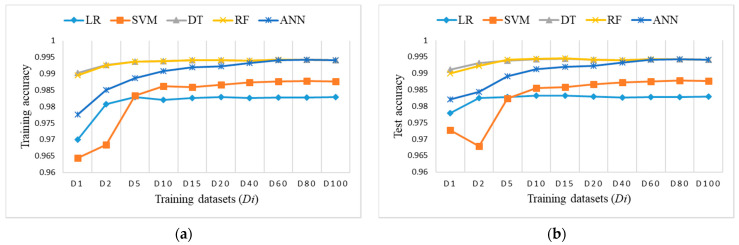
Accuracy measure on imbalanced training datasets *Di*: (**a**) training accuracy (coincidence of RF and DT); (**b**) test accuracy (coincidence of RF and DT).

Figure 9 shows accuracy results obtained on training datasets *Di* and the test dataset. DT and RF outperform others regarding the achieved accuracy and size of training datasets.

#### 3.2.2. Confusion Matrix (D20)

**Figure 10:**
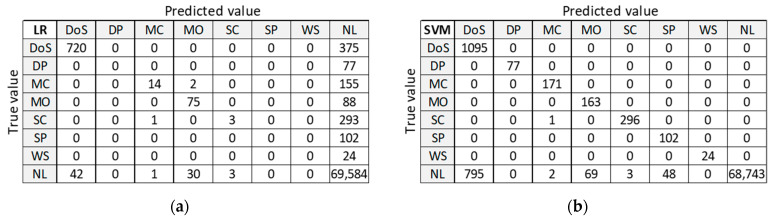

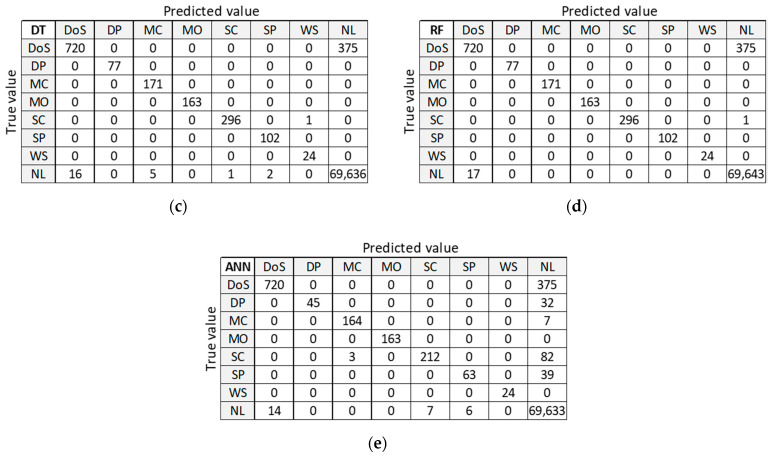
Confusion matrix on test dataset for training dataset D20: (**a**) LR; (**b**) SVM; (**c**) DT; (**d**) RF; (**e**) ANN.

Figure 10 presents classification results for training dataset D20 to be used as orientation points in the identification of the best ML algorithm on balanced datasets. LR and ANN provide good classification of the largest class ‘NL’, but misclassify many anomalies. DT and RF provide good classification for all other classes, except for class DoS with 375 misclassified samples. Only SVM algorithm correctly classifies all anomalous samples, but it performs the worst at classifying the normal class ‘NL’.

### 3.3. Evaluation of Balanced Training Datasets with Reduced Class ‘NL’

Balanced training datasets *DRi* were tested to analyze and define the smallest possible number of randomly selected samples in class ‘NL’ that gives comparable results, which are close to or even better than those from imbalanced dataset D20.

#### 3.3.1. Classification Results

**Figure 11:**
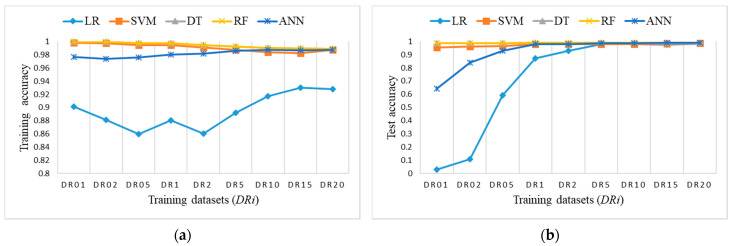
Accuracy measure for ML algorithms on balanced training datasets *DRi*: (**a**) training accuracy (coincidence of RF, SVM, and DT); (**b**) test accuracy (coincidence of RF and DT).

Figure 11 shows the accuracy results of training balanced datasets *DRi* and the test dataset for all ML algorithms. LR has the lowest accuracy for training datasets; it starts very low, but it increases rapidly and starts catching up to the accuracy of others on test dataset for DR5 and higher. A similar conclusion stands for ANN, only with higher accuracy results. Other algorithms perform well and provide comparable results on all training datasets.

#### 3.3.2. Confusion Matrix (DR5)

**Figure 12:**
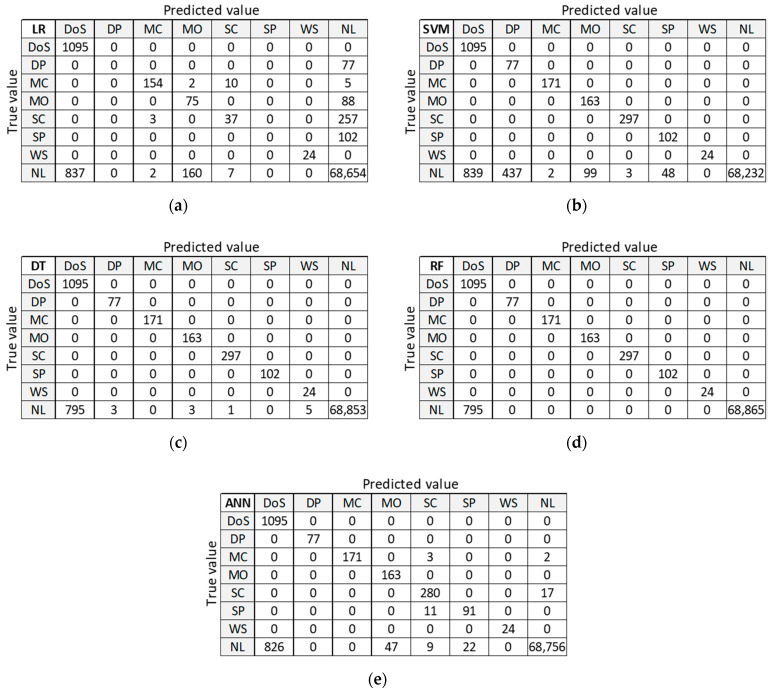
Confusion matrix on test dataset for training dataset DR5: (**a**) LR; (**b**) SVM; (**c**) DT; (**d**) RF; (**e**) ANN.

Figure 12 presents classification results for training dataset DR5 with the best accuracy on the test dataset. LR provides good classification of samples in the largest class ‘NL’, but misclassifies a large number of samples in anomalous classes (five out of seven classes). SVM, DT, and RF provide correct classifications for all anomalous classes, except for class DoS. ANN misclassifies a small number of samples in two anomalous classes and gives similar results in case of class ‘NL’.

### 3.4. Evaluation of Balanced Datasets Determined with Clustering

Balanced training datasets *DCi* were tested to analyze the approach of also minimizing anomalous classes and to define the smallest training dataset that gives comparable results, which are close to or even better than those from imbalanced dataset D20.

#### 3.4.1. Classification Results

**Figure 13:**
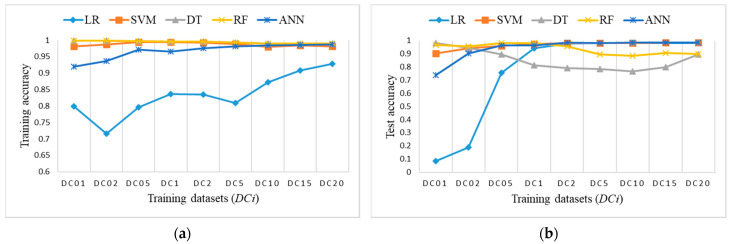
Accuracy measure for ML algorithms on balanced training datasets *DCi*: (**a**) training accuracy (coincidence of RF and DT); (**b**) test accuracy.

Figure 13 shows the accuracy results of training balanced datasets *DCi* and the test dataset for all ML algorithms. LR has the lowest accuracy for training datasets, it starts very low but it increases rapidly and starts catching up to the accuracy of others on test dataset for DC2 and higher. A similar conclusion stands for ANN, only with higher accuracy results. Other algorithms perform well and provide comparable results on all training datasets.

#### 3.4.2. Confusion Matrix (DC5)

**Figure 14:**
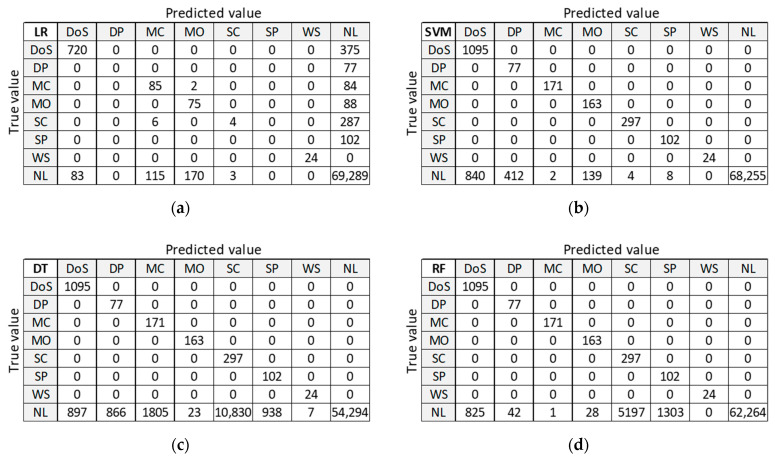

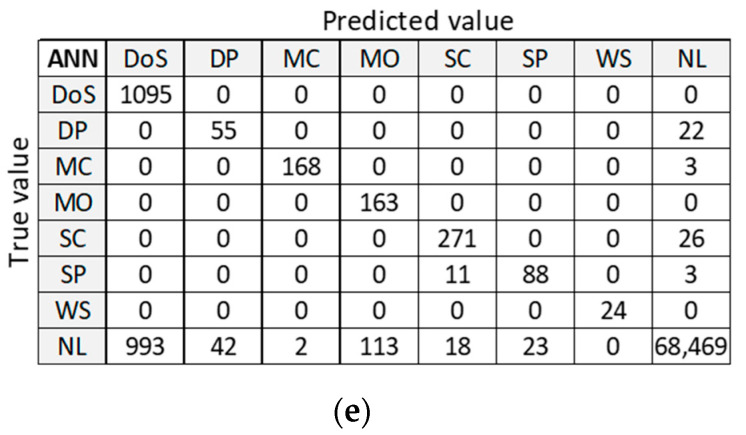
Confusion matrix on test dataset for training dataset DC5: (**a**) LR; (**b**) SVM; (**c**) DT; (**d**) RF; (**e**) ANN.

Figure 14 presents classification results for training dataset DC5 with the best accuracy on the test dataset. LR provides good classification of samples in the largest class ‘NL’, but misclassifies a large number of samples in anomalous classes (five out of seven classes). SVM, DT, and RF provide correct classifications for all anomalous classes, except for class DoS. ANN misclassifies a small number of samples in two anomalous classes and gives similar results in case of class ‘NL’.

By analyzing the two confusion matrices of the ML algorithms for datasets DR5 (Figure 12) and DC5 (Figure 14), we see that they are quite comparable. The LR algorithm is slightly better with DR5, where the main difference is the classification of ‘DoS’ class. The SVM algorithm results are very similar in both cases, except for ‘NL’ class, which is slightly worse classified than ‘DP’ and ‘SP’ classes in DR5. DT, RF, and ANN algorithms give a better classification with dataset DR5 for all classes, except for misclassifications of class ‘NL’ as class ‘DoS’ class.

### 3.5. Comparison of ML Algorithms

**Figure 15:**
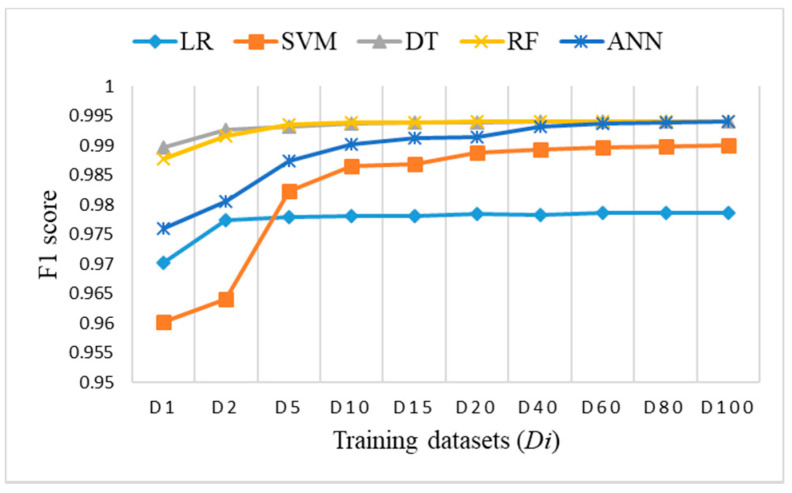
F1 score on test dataset for imbalanced datasets *Di*.

**Figure 16:**
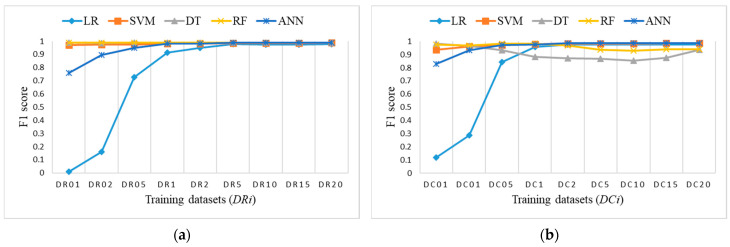
F1 score on test dataset for: (**a**) Balanced datasets *DRi* with reduced class ‘NL’ (coincidence of RF and DT); (**b**) balanced sets *DCi* with clustering.

To perform a comparison of the classification results for imbalanced datasets versus the two proposed approaches with small balanced datasets, we decided to use the F1 score, which was very similar to accuracy, but provides a better metric for further analysis. Figure 15 shows the F1 score obtained on the test dataset for training datasets *Di*. Figure 16 shows the F1 score obtained on the test dataset for training datasets *DRi* and *DCi*. In both cases, the number of samples is the same, lower than, or equal to 20% of the training dataset.

**Figure 17:**
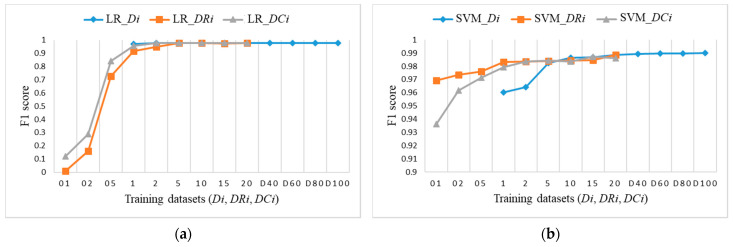
F1 score on test dataset for training datasets *Di*, *DRi*, *DCi*: (**a**) LR; (**b**) SVM.

**Figure 18:**
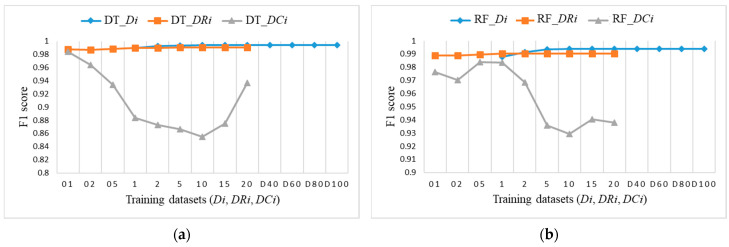
F1 score on test dataset for training datasets *Di*, *DRi*, *DCi*: (**a**) DT; (**b**) RF.

**Figure 19:**
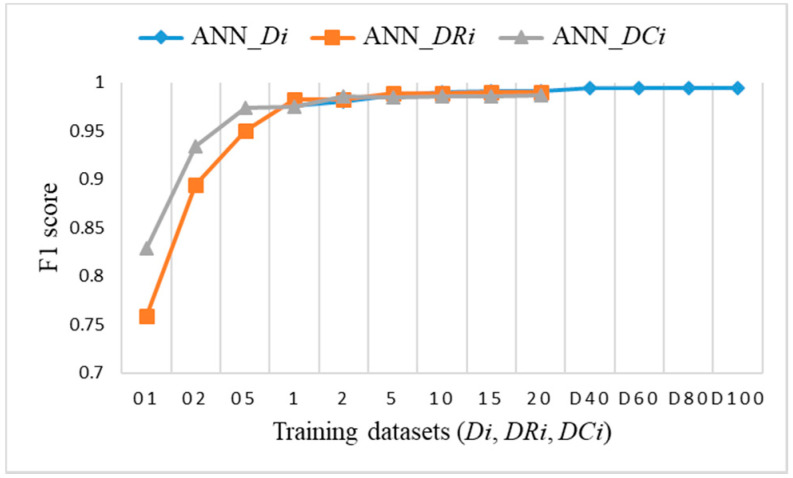
F1 score on test dataset for training datasets *Di*, *DRi*, *DCi* for ANN.

To explore the evaluation results on balanced datasets and compare them, Figure 17, Figure 18 and Figure 19 show the F1 score for each individual ML algorithm on the test dataset for training datasets *Di*, *DRi*, and *DCi*. LR, SVM, and ANN perform well on datasets from DR5 and DC5 on, while DT and RF already give comparable classification results to D1 on smaller datasets DR01, but they completely fail in the case of datasets *DCi*.

**Figure 20:**
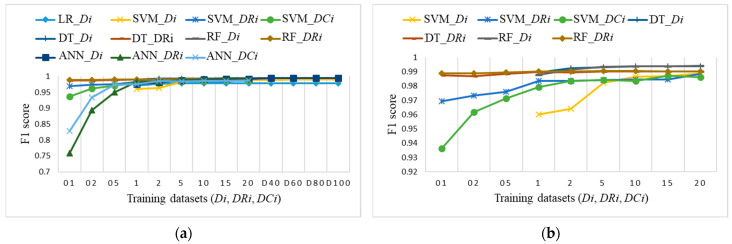
F1 score on test dataset for training datasets *Di*, *DRi*, and *DCi*: (**a**) ML algorithms for most of datasets, without LR_*DRi*, DT_*DCi*, and RF_*DCi* that are not suitable for small datasets (coincidence of LR_*Di*, DT_*Di*, DT_*DRi*, RF_*Di,* RF_*DRi,* and ANN_*Di*); (**b**) SVM, DT, and RF for smaller datasets up to 20% of the training dataset (coincidences of: DT_*DRi* and RF_*DRi*; DT_*Di* and RF_*Di*).

Figure 20a shows the F1 score for all datasets *Di*, *DRi*, and *DCi* to identify a suitable ML algorithm, the selection of training datasets and number of samples. All ML algorithms, except LR on balanced datasets, perform well on training datasets from D5, DR5, and DC5 forward. For smaller balanced training datasets *DRi*, only DT and RF perform close to the expected evaluation results, as shown in Figure 20b. SVM already provides a comparable F1 score to randomly determined dataset D10 for balanced datasets DR1 and DC2.

### 3.6. Edge Computing Results on Raspberry Pi 4

For the experimental study of edge computing, we used Raspberry Pi 4 (RPi4) [38], which is one of the most popular IoT platforms. This compact single-board computer is based on a Broadcom BCM2711 chip with Quad core Cortex-A72 (ARM v8) 64-bit system, has 1 GB SDRAM, and is equipped with a 40-pin GPIO (General Purpose I/O) connector for interfacing with external sensors. RPi4 runs a full-fledged Linux-based operating system called Raspberry Pi OS which is stored on a 64 GB micro SD memory card, as there is no internal storage available. RPi4 also does not include any special hardware for accelerating ML tasks. However, since it runs a full Linux OS, it is capable of running the same programs as more powerful computers, as long as they are compiled for an Arm instruction set. This allowed us to run the same Python scripts and use the same libraries already used on less resource-constrained devices.

#### 3.6.1. Training Time

For each ML algorithm, the Python program was run five times to measure average training time and test time for datasets up to 20% of training samples determined from the training dataset. Larger datasets were very slow, and they are not expected to be used on edge devices. After the last loop, the trained model was kept to be used for the prediction of new samples in the test dataset. The implementation of ANN, used in the present Python code, was not applicable, as the algorithm was frequently killed by the Linux kernel due to the low amount of available memory.

**Figure 21:**
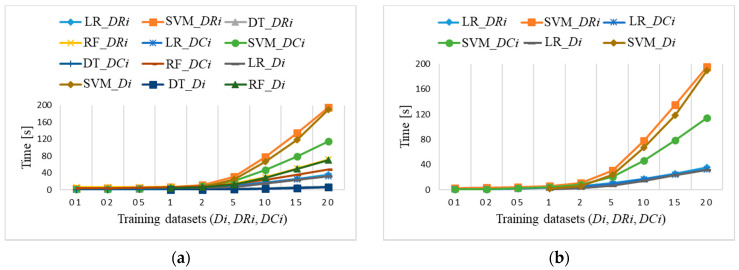
Raspberry Pi 4 training time for: (**a**) *Di*, *DRi*, *DCi* (coincidences of: RF_*Di* and RF_*DRi*; DT_*Di*, DT_*DRi*, and DT_*DCi*; LR_*Di*, LR_*DRi*, and LR_*DCi*); (**b**) LR and SVM for *Di*, *DRi*, *DCi* (coincidence of LR_*Di*, LR_*DRi*, and LR_*DCi*).

Figure 21a shows average training times for imbalanced datasets *Di* and balanced datasets *DRi* and *DCi*. They start increasing for datasets larger than 2% for three algorithms (SVM, RF, and LR), but they hardly change for the DT algorithm from 1 to 6 s for the largest dataset. However, *Di* and *DRi* datasets require larger training time as *DCi*. The SVM algorithm is the most time consuming in all cases (Figure 21b), except for *DCi* datasets being faster than the other two. Training times are the same for the LR algorithm. Standard deviations of the presented averaged results increase from 0.001 to 0.5, depending on the size of the dataset and training algorithm. Prediction includes only output computation times, which takes about 1 s for all ML algorithms.

#### 3.6.2. Memory Usage

The memory usage on Raspberry Pi 4 depends on the size of datasets and ML algorithms. In the case of large datasets, it would be necessary to define the pre-processing methods and select the suitable algorithm in advance. Based on the evaluation of small imbalanced datasets *Di*, and balanced datasets *DRi* and *DCi*, we track memory usage with the standard Python library *resource* [39]. The function *max_usage* consists of a program call *resource.getrusage().ru_maxrss*, which indicates the maximum amount of memory that is currently allocated by the process, as reported by the host operating system. For each run of ML algorithm and selected dataset, every 10 ms, memory usage is measured and finally the maximum memory allocation is returned to be evaluated.

**Figure 22:**
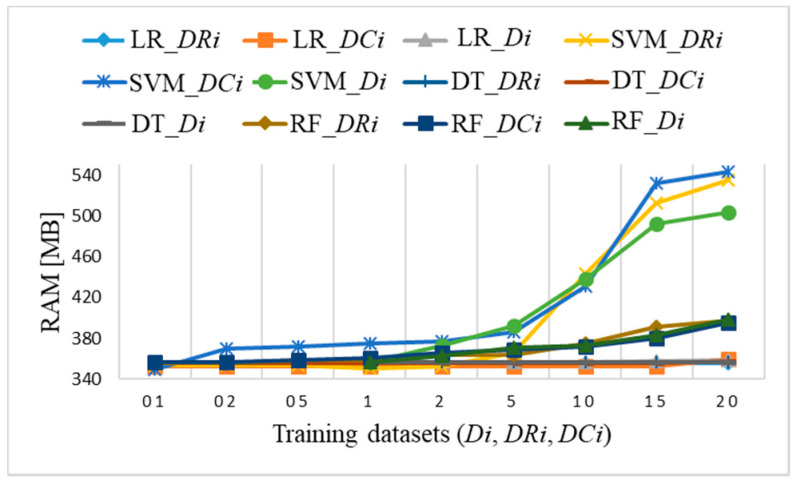
Raspberry Pi 4 memory usage for datasets *Di*, *DRi*, *DCi* with coincidence of LR and DT algorithms RAM usage of 360 MB and with coincidence of RF algorithm with RAM usage for datasets larger than D1, DR1, and DC1 up to 390 MB.

Figure 22 shows memory usage when running a Python program for all three datasets *Di*, *DRi*, and *DCi*. The allocated amount of RAM increases for two ML algorithms (SVM, RF) for subsets sizes larger than 5% of samples. The SVM algorithm is the most consuming while it requires more than 500 MB of RAM for datasets DR15 and DR20. Minor differences in memory usage occur with certain data but do not exceed 30 MB, which does not affect the performance itself. The other two ML algorithms (LR, DT) require the same amount of RAM, approximately 360 MB, independently of dataset size.

Additionally, the prediction on a test set requires an additional amount of RAM for all ML algorithms, up to 5%. The exact measurement of memory usage is a complex task influenced by various factors, e.g., operating system, program run-time environment, data structures used by algorithms, and others. For the purpose of this study, the presented results of the memory usage are rather preliminary and will be further explored with the use of Raspberry Pi 4 in edge computing for extended data analysis, pre-processing, and machine learning operations.

## 4. Conclusions

This paper presents an evaluation of the results of machine learning algorithms on a DS2OS imbalanced dataset to identify anomalies. It is obvious that the smaller balanced datasets give comparable results to the larger unbalanced datasets for all evaluation metrics. The proposed approach is first based on all samples from anomalous classes and the elimination of samples from the largest class ‘NL’. Afterwards, in the second approach, the clustering method defines datasets with representative samples from all classes in case of smaller datasets (DC01, DC02, DC05, DC1) and datasets with all samples from anomalous classes and samples from computed clusters of class ‘NL’ (DC2, DC5, DC10, DC15, DC20).

Not all examined ML algorithms provide satisfactory prediction results, which was shown in confusion matrixes, as either anomalous classes or normal class ‘NL’ are misclassified. In the evaluation of ML performance on *Di* datasets, the misclassifications generally occur for anomalous classes, while on *DRi* and *DCi* datasets, the misclassifications almost exclusively occur for the normal class ‘NL’, due to its most under-sampled cases. The most promising and acceptable solution for edge computing is the DT algorithm, which provides similar results for F1 score, while having low resource consumption and fast runtime performance on the smallest dataset DR01. Thus, we can conclude that edge computing can be a suitable alternative to cloud solutions. Analysis of a problem domain is required in order to select the best evaluation methods and ML algorithms, without too much of an impact on the desired performance results as shown in evaluation results of training time and memory usage on Raspberry Pi 4.

For the future, we plan to use more large imbalanced datasets, preprocessing, and optimization methods, and adopt several ML algorithms to be implemented as incremental learning in edge computing. The determination of smaller datasets, comparable or better classification, shorter execution times, and lower memory consumptions will be investigated and tested, especially for the additional resource constrained IoT devices.

## Figures and Tables

**Table 1:** Random distribution of DS2OS dataset to training/test dataset and subsets of training dataset (D1, D2, D5, D10, D15, D20, D40, D60, D100).

Dataset	Anomalous Data	Normal Data	Total
Original dataset (DS2OS)	10,017	278,264	357,941
Training dataset (80%)	8088	278,264	286,352
Test dataset (20%)	1929	69,660	71,589
D1 (1%)	102	2761	2863
D2 (2%)	179	5548	5727
D5 (5%)	410	13,908	14,318
D10 (10%)	842	27,793	28,635
D15 (15%)	1220	41,732	42,952
D20 (20%)	1612	55,658	57,270
D40 (40%)	3224	111,316	114,540
D60 (60%)	4831	166,980	171,811
D80 (80%)	6456	222,625	229,081
D100 (Training dataset)	8088	278,264	286,352

**Table 2:** Subsets of classes with anomalous data and percentage of samples from class ‘NL’ with normal data.

Dataset	Anomalous Data	Normal Data	Total
DR01 (0.1%)	8088	278	8366
DR02 (0.2%)	8088	557	8645
DR05 (0.5%)	8088	1391	9479
DR1 (1%)	8088	2783	10,871
DR2 (2%)	8088	5565	13,653
DR5 (5%)	8088	13,913	22,001
DR10 (10%)	8088	27,826	35,914
DR15 (15%)	8088	41,740	49,828
DR20 (20%)	8088	55,653	63,741

**Table 3:** Subsets of clustered samples from each class.

Dataset	Anomalous Data	Normal Data	Total
DC01	1770	256	2026
DC02	3061	535	3596
DC05	4834	1395	6229
DC1	6266	2815	9081
DC2	8088	5656	13,744
DC5	8088	14,177	22,265
DC10	8088	28,379	36,467
DC15	8088	42,580	50,668
DC20	8088	56,784	64,872

## Data Availability

The DS2OS dataset used in this study is available online from Kaggle: https://www.kaggle.com/francoisxa/ds2ostraffictraces (accessed on 20 November 2020).
